# The role of gut microbiota and drug interactions in the development of colorectal cancer

**DOI:** 10.3389/fphar.2023.1265136

**Published:** 2023-08-23

**Authors:** Jinna Wu, Cong Xia, Can Liu, Qianshi Zhang, Chenglai Xia

**Affiliations:** ^1^ Guangzhou Key Laboratory of Basic and Applied Research of Oral Regenerative Medicine, Department of Pharmacy, Guangdong Engineering Research Center of Oral Restoration and Reconstruction, Affiliated Stomatology Hospital of Guangzhou Medical University, Guangzhou, China; ^2^ Department of Gastrointestinal Surgery, The Second Affiliated Hospital of Dalian Medical University, Dalian, China; ^3^ Affiliated Foshan Maternity and Child Healthcare Hospital, Southern Medical University, Foshan, China; ^4^ School of Pharmaceutical Sciences, Southern Medical University, Guangzhou, China

**Keywords:** gut microbiota, drugs, interactions, colorectal cancer, mechanism of action

## Abstract

The human gut microbiota is a complex ecosystem regulating the host’s environmental interaction. The same functional food or drug may have varying bioavailability and distinct effects on different individuals. Drugs such as antibiotics can alter the intestinal flora, thus affecting health. However, the relationship between intestinal flora and non-antibiotic drugs is bidirectional: it is not only affected by drugs; nevertheless, it can alter the drug structure through enzymes and change the bioavailability, biological activity, or toxicity of drugs to improve their efficacy and safety. This review summarizes the roles and mechanisms of antibiotics, antihypertensive drugs, nonsteroidal anti-inflammatory drugs, lipid-lowering drugs, hypoglycemic drugs, virus-associated therapies, metabolites, and dietary in modulating the colorectal cancer gut microbiota. It provides a reference for future antitumor therapy targeting intestinal microorganisms.

## 1 Introduction

There are over 193 million cases of colorectal cancer (CRC) diagnosed each year in the world, despite the rising use of colonoscopies (Siegel et al., 2023). Approximately 50%–60% of incident cases of CRC can be attributed to modifiable risk factors such as smoking, drinking a lot of alcohol, being overweight, being obese, being inactive, eating much red and processed meat, and eating little whole grains or fiber (Li et al., 2022). A growing body of evidence indicates that intestinal microbiomes are influenced by the environment and contribute to disease (Sepich-Poore et al., 2021; Xia et al., 2023a; Xia et al., 2023b). It has been found that patients with CRC have different intestinal microbiomes compared to healthy individuals. Moreover, it has been shown that gut microbes change during colorectal carcinogenesis assisting in identifying individuals at risk (Hagan et al., 2019). People have learned a great deal about the composition of the human gut microbiota in the last decade, but the complex temporally spatial interplay between gut microbes and humans remains an ongoing mystery.

It is well-known that the gut microbiota can be shifted into alternative stable states or quasi-stable states by antibiotics, which may become more resilient to external influences (Hao et al., 2020; Reyman et al., 2022). However, a bidirectional relationship exists between intestinal flora and non-antibiotic drugs. Despite its impact on drugs, it can also alter the structure of drugs via enzymes, enhance the bioavailability and biological activity of drugs, or reduce their toxicity (de Gunzburg et al., 2018; Willmann et al., 2019). There have also been new changes in the treatment of tumors (Ye et al., 2023). In this review, we summarize the roles and mechanisms of antibiotics, antihypertensive drugs, nonsteroidal anti-inflammatory drugs, lipid-lowering drugs, hypoglycemic drugs, virus-associated therapies, metabolites, and dietary in modulating the CRC gut microbiota. It provides evidence that intestinal microorganisms could be used as targets for future antitumor therapies.

## 2 Drug repurposing and microbiota in colorectal cancer

Drug repurposing involves using existing drugs to treat diseases not included in the original indication. Based on the side effects of the current chemotherapeutic agents, the repurposing of noncancer drugs in the prevention or treatment of CRC has the advantage of a high safety level and fewer side effects. Currently, the relationship between the gut microbiome and CRC is gradually being elucidated (Karpiński et al., 2022), and we have summarized the anticancer activity of some traditional drugs and attempted to analyze their association with the gut microbiome during treatment.

### 2.1 Antihypertensive drugs

Hypertension is the most prevalent chronic cardiovascular disease globally and is closely associated with the gut microbiota. Human global gut microbiota diversity is associated with hypertension (Silveira-Nunes et al., 2020; Wang et al., 2021). The gut microbiota is high in *Proteobacteria* and *Actinobacteria* at a low level; at the genus level, *Klebsiella*, *Clostridium*, *Streptococcus*, *Parabacteroides*, *Eggerthella*, and *Salmonella* are more prevalent (Yan et al., 2017). The proportion of butyrate decreased (Wang et al., 2021). Short-chain fatty acids (SCFA), including butyrate, regulate the activity of G protein-coupled receptors and these metabolites have immunomodulatory functions. They also contribute to blood pressure homeostasis and are implicated in the pathogenesis of hypertension (Katsi et al., 2019; Duttaroy, 2021). Meanwhile, trimethylamine, trimethylamine-producing bacteria, and trimethylamine-N-oxide have been linked to hypertension in several pathways (Zhang et al., 2021).

In animal studies, the ratio of *Firmicutes* to *Bacteroidetes* in the hypertensive model rats was increased (Santisteban et al., 2017; Li et al., 2021). Prehypertension and hypertension are associated with the risk of multiple cancers, including CRC (Seretis et al., 2019; Lee et al., 2020). Both elevated systolic and diastolic blood pressure and stage 2 hypertension were positively related to the CRC risk, and metabolic syndrome was associated with an increased risk of early-onset CRC, including hypertension (Chen et al., 2021; Kaneko et al., 2021).

**FIGURE 1:**
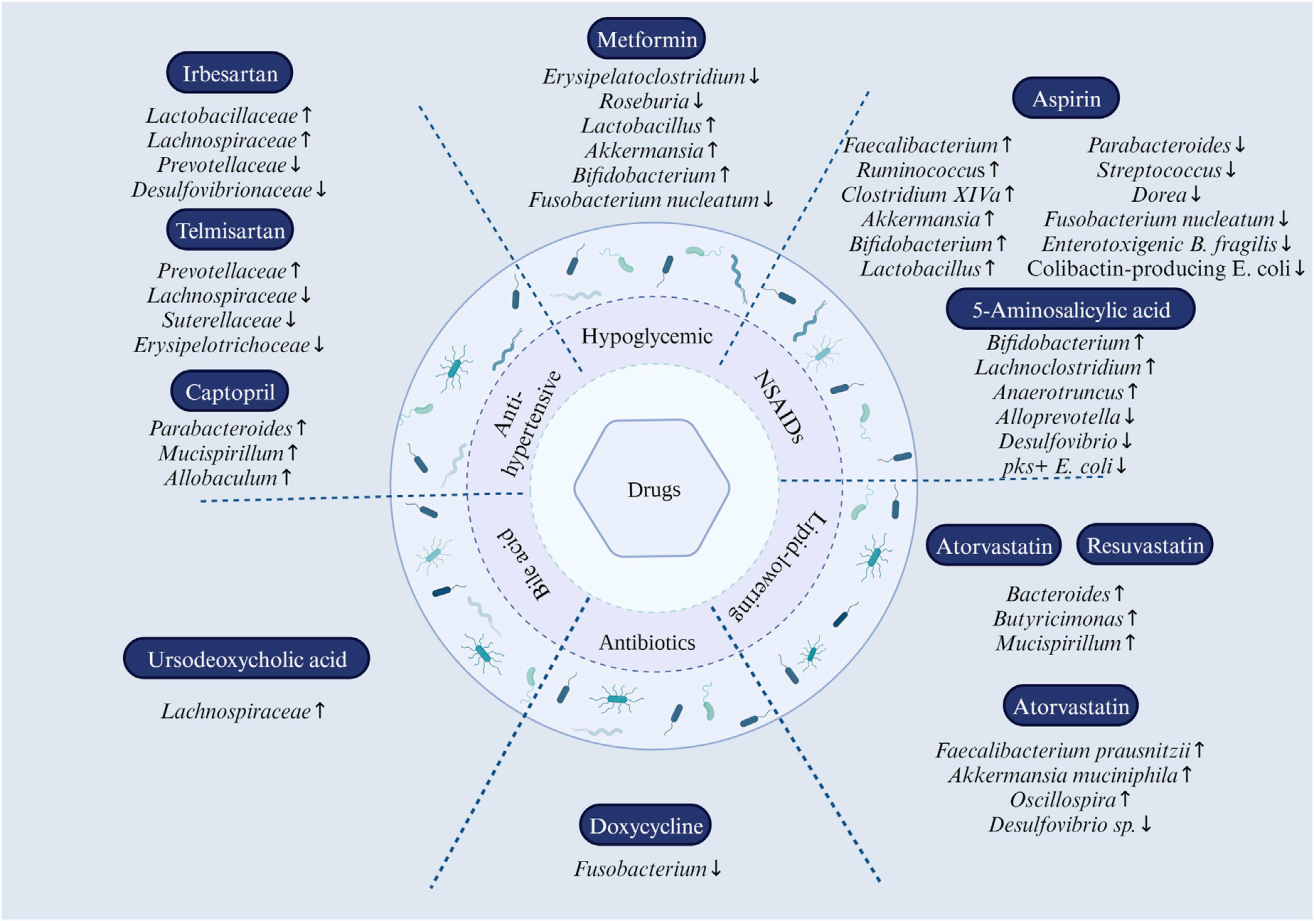
Effect of drug reuse on intestinal microbiota. In antihypertensive drugs, *Prevotellaceae* were elevated, and *Lachnospiraceae*, Suterellaceae, and *Erysipelotrichoceae* were decreased by the use of telmisartan; irbesartan upregulates the abundance of *Lactobacillaceae* and *Lachnospiraceae* and downregulates the abundance of *Prevotellaceae* and *Desulfovibrionaceae*; captopril modulates the abundance of *Parabacteroides*, *Mucispirillum*, and *Allobaculum*. In NSAIDs, aspirin increases the abundance of *Faecalibacterium*, *Ruminococcu*s, *Clostridium XIVa*, *Akkermansia*, *Bifidobacterium*, and *Lactobacillus*; while reducing the abundance of *Parabacteroides*, *Streptococcus*, *Dorea*, *Fusobacterium nucleatum*, enterotoxigenic *B. fragilis*, and colibactin-producing *E. coli. 5-Aminosalicylic acid modulates intestinal flora dysbiosis by increasing the abundance of Bifidobacterium, Lachnoclostridium, and Anaerotruncus and decreasing the abundance of Alloprevotella and Desulfovibrio.* In statins, atorvastatin and rosuvastatin upregulated the abundance of *Bacteroides*, *Butyricimonas*, and *Mucispirillum*; atorvastatin helps to reshape the dysbiosis of the gut microbiota by increasing the abundance of anti-inflammatory bacteria such as *Faecalibacterium prausnitzii*, *Akkermansia muciniphila*, and the genus *Oscillospira*, and decreasing the abundance of pro-inflammatory bacteria *Desulfovibrio* sp. Metformin treatment altered the composition of the gut microbiota by increasing the relative abundance of *Lactobacillus* and *Akkermansia* species while reducing *Erysipelatoclostridium*. At the genus level, *Bifidobacterium* increased and *Fusobacterium nucleatum* decreased. Doxycycline eliminated *Fusobacterium spp*. Members of the *Lachnospiraceae Family* (*Phylum Firmicutes*, *Class Clostridia*) were significantly increased in the gut microbial community of the ursodeoxycholic acid-treated group.

Drugs for hypertension include angiotensin I-converting enzyme inhibitors (ACEI), angiotensin II receptor blockers (ARB), and β-blockers. Angiotensin II differential blood pressure was regulated by gut microbiota metabolites. Mice lacking gut microbiota are protected from angiotensin II-induced arterial hypertension, vascular dysfunction, and hypertension-induced end-organ damage (Karbach et al., 2016). ACEI, ARB, and β -blockers significantly affect gut microbiota (Zhernakova et al., 2016; Jackson et al., 2018). Among them, telmisartan induces specific gut microbiota characteristics to mediate its anti-obesity effect; irbesartan attenuates pulmonary arterial pressure in the high-altitude pulmonary hypertension model rats by increasing the abundance of *Lactobacillaceae* and *Lachnospiraceae* in the gut and reducing the abundance of *Prevotellaceae* and *Desulfovibrionaceae*; captopril exerts its sustained antihypertensive effect by mediating captopril-reactive bacteria (including *Parabacteroides*, *Mucispirillum*, and *Allobaculum*) (Figure 1), and re-balancing of the brain-gut axis; Antihypertensive peptides and the α-lactalbumin hydrolysates under 3 kDa can restore the diversity of the intestinal microbiota, induce SCFA, and relieve hypertension-associated gut dysbiosis (Yang et al., 2019; Beckmann et al., 2021; Nijiati et al., 2021; Xie et al., 2022).

These hypertensive drugs also have a preventive or therapeutic effect on CRC. Some studies demonstrated that the application of ACEI and ARB might reduce CRC incidence, polyp formation, and metastasis and prevent the development of CRC via a mechanism involving inhibition of angiotensin-converting enzyme activity, angiotensin Ⅱ synthesis, and the epidermal growth factor receptor expression (Kedika et al., 2011; Makar et al., 2014; Childers, 2015; Asgharzadeh et al., 2018; Nakamura et al., 2018;Cheung et al., 2020a). ARB did not change the number of cluster of differentiation 11 b + bone marrow cells in tumors; nonetheless, it significantly reduced the T cell inhibition ability while reducing the production of various immunosuppressive factors. ARB also decreased the cancer-related fibroblasts, chemokine ligand 12, and nitric oxide synthase 2 expressions, indicating that the renin-angiotensin-system is involved in the production of immunosuppressive cells initiated by bone marrow cells and fibroblasts tumor microenvironment (Nakamura et al., 2018). ACEI/ARB and β-blockers are associated with improved survival, tumor progression, and decreased hospitalization in patients with advanced CRC (Engineer et al., 2013). The β-blocker nebivolol co-inhibited complex I and adenosine triphosphate synthase activity, precisely hindering oxidative phosphorylation of cancer cells (Nuevo-Tapioles et al., 2020). Antihypertensive medications regulate intestinal flora and maintain a healthy brain-gut axis. Angiotensin II regulates blood pressure through gut microbiota metabolites, thereby involving intestinal flora participation in the renin-angiotensin-system and the formation of the suppressive tumor microenvironment.

### 2.2 Nonsteroidal anti-inflammatory drugs

Nonsteroidal anti-inflammatory drugs (NSAIDs) can reduce the incidence and recurrence of advanced colorectal adenomas and CRC, and numerous randomized, controlled, and double-blind clinical trials are undergoing to evaluate NSAIDs in cancer chemoprevention (Wang et al., 2018; Cheung et al., 2020b; Chen et al., 2021; Chudy-Onwugaje et al., 2021; Maniewska and Jeżewska, 2021). NSAIDs play an anti-inflammatory role by inhibiting the cyclooxygenase enzyme, prostaglandin E2 pathway, and cyclooxygenase-independent pathway. The inflammatory response and accessory inflammation, which is an intermediate state between chronic inflammation and basal homeostasis, significantly impacted CRC progression and p53 to maintain homeostasis (Ghanghas et al., 2016). Upon loss of p53, para-inflammation loses its tumor-suppressive properties and becomes tumor-promoting (Lasry et al., 2016). Phosphatidylinositol-4,5-bisphosphate 3-kinase catalytic subunit alpha mutations are present in approximately 15% to 20% of CRC. Upregulation of phosphatidylinositol 3-kinase increases cyclooxygenase-2 activity, promotes prostaglandin E2 synthesis, and inhibits apoptosis in colon cancer cells (Amitay et al., 2019). NSAIDs can also inhibit the mechanistic target of rapamycin kinase signaling to induce autophagy, inhibit tumor cell viability via p53-dependent autophagy, and increase chemotherapeutic drug radiosensitivity and cytotoxicity (Yu et al., 2018). Patients using NSAIDs have distinct gastrointestinal microbiome profiles compared to those not using NSAIDs. The abundance of *Prevotella spp.*, *Bacteroides spp.*, family *Ruminococaceae*, and *Barnesiella spp.* can discriminate aspirin users from no medication; celecoxib and ibuprofen users showed enrichment of *Acidaminococcaceae* and *Enterobacteriaceae* in the gut (Rogers and Aronoff, 2016).

Aspirin has potent chemopreventive activity in CRC and regular use of aspirin significantly reduces the incidence of CRC (Bosetti et al., 2020). The incidence of CRC is significantly reduced in people with long-term aspirin use for more than 10 years (Zhang et al., 2021). Low-dose aspirin and flexible sigmoidoscopy are equally effective in reducing the incidence and mortality of CRC (Emilsson et al., 2017). Kane, A.M. et al. used the B-Raf proto-oncogene mutation model study and found that aspirin treatment could significantly reduce the incidence of metastatic disease (Kane et al., 2021). The 10-year overall survival was significantly prolonged with aspirin in patients with wild-type phosphatidylinositol-4,5-bisphosphate 3-kinase catalytic subunit alpha and mutated KRAS tumors (Gebauer et al., 2021). The chemopreventive effect of aspirin in CRC might be related to various mechanisms, including inhibition of cyclooxygenase, cyclin-dependent kinase, β-catenin phosphorylation, mechanistic target of rapamycin kinase, MYC, cyclin A2, nuclear factor of kappa light polypeptide gene enhancer in B cells, and Wnt signaling pathways, activation of adenosine monophosphate kinase, induction of polyamine catabolism and deoxyribonucleic acid mismatch repair proteins, and acetylation of p53, glucose-6-phosphate dehydrogenase, and other proteins (Sankaranarayanan et al., 2020).

It has been found that aspirin can regulate the tumor microenvironment via gut microbes, induce autophagy, reduce the inflammatory response induced by necrosis, and inhibit tumor development (Frouws et al., 2017). Aspirin alters the gut microbiome and modifies the bacterial taxa in the gut, thereby protecting against CRC (Chan et al., 2012). Specifically, aspirin downregulated flora positively associated with CRC risk, including *Parabacteroides* and *Streptococcus*, while up-regulating flora negatively associated with CRC, including *Faecalibacterium* and *Ruminococcus* (Figure 1). In double-blind, randomized, placebo-controlled trials, aspirin users have a higher abundance of *Ruminococcaceae* and *Clostridium XIVa* and a lower abundance of *Parabacteroides* and *Dorea*. In addition, aspirin increased the abundance of the anti-inflammatory bacterium *Akkermansia* more than the placebo (Figure 1) (Prizment et al., 2020; Brennan et al., 2021). Likewise, the inhibitory effect of aspirin on the notorious *Fusobacterium nucleatum*, enterotoxigenic *Bacteroides fragilis*, and colibactin-producing *Escherichia coli* is noteworthy for its ability to diminish *F. nucleatum* on CRC promotion (Figure 1). In mice treated with azomethane and dextran sulfate, probiotic bacteria, including *Bifidobacterium pseudolongum* and *Faecalibacterium rodentium*, were enriched in aspirin-treated mice; a similar effect also occurs for *Bifidobacterium* and *Lactobacillus genera*, including *B. pseudolongum*, *Bifidobacterium breve*, *Bifidobacterium animalis*, *Lactobacillus reuteri*, *Lactobacillus gasseri*, and *Lactobacillus johnsonii* in aspirin-treated APCmin/+ mice (Figure 1) (Zhao et al., 2020).

Celecoxib is a Food and Drug Administration-approved drug used for colorectal polyps and prevents high-risk adenoma recurrence (Lynch et al., 2010; Thompson et al., 2016). In a 5-year clinical trial designed to prevent sporadic adenomatous polyps in colon, the incidence of new adenoma was substantially lower in the celecoxib group than in the placebo group. Celecoxib reduced the luminal microbiome and metabolome associated with intestinal stem cell proliferation, inhibited c-Met, and exerted anti-CRC effects (Jendrossek, 2013; Lin et al., 2019). The ability of celecoxib to chemoprevent CRC is mediated by the gut microbiota and microbe-derived metabolites (Ferrara et al., 2022). In hepatocellular carcinoma, celecoxib enhances the anti-tumor function of immune cells by up-regulating the abundance of *Bacteroides Acidifaciens*, *Odoribacter Laneus,* and *Odoribacter splanchnicus* (Pan et al., 2023).

5-Aminosalicylic acid has also been found to exert anti-inflammatory effects by modulating the intestinal flora. 5-Aminosalicylic acid modulates intestinal flora dysbiosis by increasing the abundance of *Bifidobacterium*, *Lachnoclostridium*, and *Anaerotruncus* and decreasing the abundance of *Alloprevotella* and *Desulfovibrio* (Figure 1). 5-Aminosalicylic acid is an important metabolite of aspirin and has been found to exert anti-inflammatory effects by modulating intestinal flora. abundance to modulate intestinal dysbiosis and ultimately ameliorate dextran sulfate sodium-induced colitis (Huang et al., 2022; Wada et al., 2023). In addition, 5-aminosalicylic acid may also exert a preventive effect against CRC by inhibiting pks + *E. coli* (Figure 1) (Tang-Fichaux et al., 2021). Naproxen may influence the production of trimethylamine and trimethylamine-N-oxide by altering the type of choline-utilizing anaerobic bacteria implicated with CRC progression (Rogers and Aronoff, 2016).

Besides, intestinal flora also impacts the drug use of NSAIDs. The bioavailability of aspirin is related to its chemopreventive effect on CRC, however, the bioavailability of oral drugs, including aspirin, is related to intestinal flora (Zhang et al., 2021). *Lysinibacillus sphaericus* weakens the chemopreventive effect of aspirin by degrading it (Zhao et al., 2020). Not coincidentally, enzymes produced by the intestinal flora reduced the efficacy of 5-aminosalicylic acid in the treatment of inflammatory bowel disease (Mehta et al., 2023). Therefore, these findings reveal the complex interactions between aspirin, intestinal flora, and CRC, and provide additional mechanisms and references to follow for the use of aspirin in the treatment of CRC.

### 2.3 Lipid-lowering drugs

High serum triglyceride levels and high serum cholesterol levels are positively associated with the incidence of CRC (Yang et al., 2022). Targeting cholesterol biosynthesis, which contributes to CRC cell growth and liver metastasis, is a promising therapy for CRC (Zhang et al., 2021). As lipid-lowering agents, statins are among the chemopreventive agents for CRC and play an important role in the treatment and prognosis of CRC. A population-based case-control study found statins to be moderately chemoprotective against CRC (Rodríguez-Miguel et al., 2022). After undergoing a colonoscopy, statin users were found to have a lower risk of CRC (Cheung et al., 2019). Furthermore, atorvastatin eliminated microadenomas in tumor-free mice (Chang et al., 2018). Statins also inhibit the proliferation of CRC cells and promote apoptosis (Palko-Łabuz et al., 2019). Statins may be considered as targeted therapy for CRC (Shailes et al., 2022). Meanwhile, in the treatment, statins can enhance the anticancer activity of the chemotherapeutic drug oxaliplatin, enhance the sensitivity of radiotherapy for CRC and synergize the antitumor effect of the targeted drug regorafenib, which may arise from the weakening of stemness and drug resistance of CRC cells by statins (Karagkounis et al., 2018; Gao et al., 2021; Gonçalves et al., 2021; Tsubaki et al., 2023; Yuan et al., 2023). Pravastatin, a metabolite of intestinal flora, can promote interleukin (IL)-13 release from type Ⅱ innate lymphocytes through IL-33 signaling, which has been shown to promote self-renewal of pluripotent intestinal stem cells at the base of intestinal crypts (Zhu et al., 2019; Deng et al., 2021). In postoperative patients, statin use has been associated with lower short- and long-term mortality (Pourlotfi et al., 2021a; Pourlotfi et al., 2021b).

The gut microbiota is an important part of the pharmacological action of statins. Statin therapy is negatively associated with obesity-related microbiota dysbiosis (Vieira-Silva et al., 2020). Statins modulate gut microbiota dysbiosis due to hyperglycemia and hyperlipidemia, and atorvastatin and rosuvastatin upregulated the abundance of *Bacteroides*, *Butyricimonas*, and *Mucispirillum*, and notably, fecal transplants of gut microbiota altered by rosuvastatin still exerted ameliorative effects on hyperglycemia (Figure 1) (Kim et al., 2019). Atorvastatin helps to reshape the dysbiosis of the gut microbiota induced by hyperlipidemia, which includes reversing the ratio between *Firmicutes* to *Bacteroidetes*, increasing the abundance of anti-inflammatory bacteria such as *Faecalibacterium prausnitzii*, *Akkermansia muciniphila*, and genus *Oscillospira*, and decrease the abundance of pro-inflammatory bacteria *Desulfovibrio sp.* (Figure 1) (Wang L. et al., 2021). There is no doubt that the same microbiota regulated by statins are associated with CRC. An interesting perspective is that the gut microbiota may already be a common target for statins against atherosclerosis and tumors (Wu et al., 2021). The study found that intestinal flora is crucial for preventing or treating CRC with statins. Statins may affect the gut microbiota, with a high proportion of *Bacteroides*, a low proportion of *Faecalibacterium*, and a low microbial cell density in the gut of patients not receiving statin therapy compared with patients taking statins. Statins can enhance the number of *Anaerostipes hadrus* and *Bifidobacterium longum subsp* and their ability to produce butyrate Statins also increase the abundance of *Bifidobacterium*, *Anaerobes*, *Broucella*, and *B. longum subsp* (Figure 1) (Vieira-Silva et al., 2020; Hu et al., 2021). Statins are equally involved in the metabolism of the gut microbiota. The application of atorvastatin enhances the availability of tryptophan in the gut, which in turn leads to an increase in *L. reuteri*, which then inhibits colorectal carcinogenesis by metabolizing tryptophan to indole-3-lactic acid. This process ultimately contributes to the chemopreventive effects of atorvastatin (Figure 1) (Han et al., 2023).

### 2.4 Hypoglycemic drugs

Diabetes mellitus is associated with an increased risk of CRC, and their association is related to the family history of CRC (Ali Khan et al., 2020a; Ali Khan et al., 2020b; Amadou et al., 2021; Hu et al., 2021). High insulin levels occurring in the pre-diabetic phase act as a major driver of the positive association of type 2 diabetes with CRC (Murphy et al., 2022). The duration of obesity reflects the level and duration of hyperinsulinemia, patients with long-term obesity had an increased risk of CRC compared to patients with no history of obesity (Peeters et al., 2015). Stratification based on the cumulative period of obesity revealed that patients with long-term obesity had an increased risk of CRC compared to patients with no history of obesity. Furthermore, insulin and insulin like growth factor 1 (IGF1) are ligands for the IGF1 receptor, and their binding induces autophosphorylation and conformational changes in the cytoplasmic tyrosine domain to stimulate a signaling cascade, including primarily the phosphatidylinositol 3′-kinase/protein kinase B and mitogen-activated protein kinases pathways that are closely associated with protein synthesis, survival, and proliferation (Yu et al., 2022).

Certain hypoglycemic drugs can prevent and treat CRC. Metformin has a potential role in the chemoprevention of CRC, and low-dose metformin reduces the incidence and number of metachronous adenoma or polyps after polypectomy (Higurashi et al., 2016); Metformin, as neoadjuvant therapy, can reduce the adverse effects of diabetes and improve the prognosis of patients with diabetes and CRC in conjunction with 5-fluorouracil (Sang et al., 2020). The mechanisms include metformin targeting the mechanistic target of rapamycin kinase via the adenosine monophosphate-activated protein kinase and insulin/insulin-like growth factor pathway, and inducing apoptosis and autophagy through oxidative stress, inflammation, and metabolic homeostasis (Mallik and Chowdhury, 2018).

Studies have shown that metformin inhibits tumor progression by modifying the gut microbiome. Transplantation of metformin-treated mice’s feces into mice with metastatic tumors revealed increased SCFA and decreased expression of tumor cholesterol metabolism genes in tumor-bearing mice (Broadfield et al., 2022). Metformin can increase *Firmicutes* and reduce *Fusobacteria* and *Bacteroidetes* at the phylum level. At the genus level, there was an increased *Bifidobacterium* and decreased *Fusobacterium nucleatum* (Figure 1) (Huang et al., 2020; Huang et al., 2020). Metformin altered the composition of gut flora associated with CRC, including *Bacteroides*, *Streptococcus*, *Achromobacter*, *Alistipes*, and *Fusobacterium*. Importantly, metformin inhibited the growth of *Fusobacterium in vitro* and was shown to inhibit *Fusobacterium* in APC Min/+ mice (Figure 1) (Huang et al., 2020).

The complex mechanism of metformin action maybe 1) metformin is metabolized in the gut, reacts with intestinal microbes, alters the composition and abundance of intestinal flora, and causes a cascade related to intestinal flora, such as regulating inflammation, innate immunity, and adaptive immunity. 2) metformin alters gut metabolomics, including some substances that can weaken CRC such as SCFA (Vallianou et al., 2019). 3) complex interactions between metformin, gut microbiota, and the growth hormone/IGF-1 axis. Metformin can affect IGF-1 levels (Khan et al., 2023). Similarly, the intestinal flora can regulate IGF-1 through the growth hormone/IGF-1 axis. Microbial metabolites, including SCFA, also regulate the release of growth hormones in cancer (Yan and Charles, 2018). The gut microbiota produces SCFA and other microbial metabolites that act on the liver and adipose tissue to induce IGF-1 production (Matsushita et al., 2021). IGF-1 production is associated with CRC risk and promotes inflammation-related tumorigenesis (Youssif et al., 2018; Murphy et al., 2020).

### 2.5 Antibiotics

Antibiotics have contradictory effects on intestinal microflora. First, prolonged misuse of antibiotics, generation, and drug resistance severely disrupt the microbial ecosystem and increase the risk of CRC. Antibiotic use was associated with an increased risk of colorectal polyps, and gut dysbiosis was implicated in the early phases of colorectal carcinogenesis, according to a case-control study conducted in Sweden. Tetracycline and quinolone antibiotics are strongly linked to an elevated risk of colon and rectal polyps (Song et al., 2021). Second, chronic inflammation induced by antibiotics can cause gene mutation in colon epithelial cells and aberrant deoxyribonucleic acid methylation modifications, contributing to CRC development. In the azoxymethane/dextran sodium sulfate mouse model, antibiotics that modulate the gut microbiota can reduce colonic inflammation and inhibit colonic tumorigenesis. The study revealed that when tumor-bearing mice were treated with the *Fusobacterium*-resistant antibiotic erythromycin, neither the tumor volume nor the abundance of *Clostridium bacteria* in the tumor tissue changed; however, when the tumor-bearing mice were treated with the *Fusobacterium*-sensitive antibiotic metronidazole, both the tumor volume and the abundance of *Fusobacterium* in the tumor tissue decreased (Mihai et al., 2021). This indicates that antibiotics regulate *Fusobacterium* and inhibit CRC tumor cell proliferation. We demonstrated that the anticancer activity of doxycycline is mainly because the drug can significantly affect the diversity of *Bifidobacterium* populations, eliminating *Fusobacterium* without any effect on other intestinal bacterial populations (Figure 1) (Elvers et al., 2020).

The relationships between gut microbiota, inflammation, and tumors are complicated. Antibiotics are a double-edged sword against intestinal flora. Antibiotics can interfere with normal intestinal flora and promote tumor occurrence; they can also inhibit pathogenic intestinal bacteria, reduce inflammation and inhibit tumor occurrence. Therefore, the effect on gut microbes should be considered when investigating antitumoral antibiotics.

### 2.6 Virus-related therapy

Viruses are part of the gut microbiota and significantly affect the occurrence and progression of CRC. Compared to healthy control, CRC patients showed higher viral diversity in the gut. Enterovirus and disease stage-specific changes and prognosis were also associated with CRC patients’ prognosis (Nakatsu et al., 2018). Epstein-Barr virus, human cytomegalovirus, human papillomavirus, and other deoxyribonucleic acid viruses replicate within infected cells, target p53, pRb, and p21, and disrupt the cell cycle. Therefore, it can produce an anti-CRC effect by interfering with the virus. Zidovudine is a nucleoside antiretroviral drug inhibiting CRC cell proliferation (Fang et al., 2017; Sherif et al., 2021). Ganciclovir inhibits the NLR family pyrin domain containing 3 activations and reduces the irinotecan-induced intestinal toxicity (Huang et al., 2020). The combination of zidovudine and veavirren completely blocked tumorigenesis in tumor-bearing mice (Schneider et al., 2021). Nelfinavir is a human immunodeficiency virus protease inhibitor and fractionated radiotherapy for locally advanced rectal cancer (Hill et al., 2016). Chemokine receptors C-X-C motif chemokine receptor 4 and C-X-C motif chemokine receptor 7 are involved in CRC progression, and HIV drugs targeting C-X-C motif chemokine receptor 4 may inhibit CRC (Goïta and (Goïta and Guenot, 2022).

Studies have shown that double-stranded DNA viruses in the gut are mainly phages (Hannigan et al., 2018; Lin and Lin, 2019). The abundance of phages was significantly higher in azoxymethane-induced colorectal tumors. It disrupts the intestinal microbiota balance and induces a specific immune response that exacerbates colitis through the toll-like receptor 9 and interferon-gamma pathways. Because antibiotics are often difficult to regulate the intestinal flora precisely and can interfere with normal intestinal flora, bacteria will develop gradual antibiotic resistance. Thus, antitumor therapies targeting phages have distinct advantages. Bakuradze et al. found that adding phage VA7 to enterotoxin-producing *Bacteroides fragilis*-infected CRC cells significantly decreased bacterial counts and IL-8 levels in the VA7-treated group compared to untreated infected cells (Bakuradze et al., 2021). Bacteriophage FNU 1 can decompose, lyse *F. nucleatum* biofilms, and play an antitumor role (Kabwe et al., 2019). Bacteriophage EFA 1 upregulates reactive oxygen species in the culture system and inhibits HCT116 CRC cell growth (Kabwe et al., 2021).

It is possible to obtain phage nanoparticles with increased antibacterial activity through phage display technology by selecting phages with a high affinity for the target and combining them with inorganic nanomaterials having elevated antibacterial activity. For instance, the incorporation of silver nanoparticles into the phage surface resulted in precise clearance of *Fusobacterium*, blocked the expansion of bone marrow-derived suppressor cells in the tumor microenvironment, enhanced the host immune response, and prolonged the survival of CRC mice (Dong et al., 2020). Oral administration of phage nanoparticles containing irinotecan did not affect the number of blood cell levels, immunoglobulin and histamine levels, and liver and kidney function of tumor-bearing animals indicated that the safety of phage nanoparticles was good (Zheng et al., 2019). Phages can carry different fragments and have different effects on the tumor microenvironment. It was found that bacteriophage M13, which targets carcinoembryonic antigen, can specifically bind to the carcinoembryonic antigen in tumor cells, activate antitumor immunity, and substantially inhibit tumor growth in CRC tumor-bearing mice (Murgas et al., 2018).

Oncolytic viruses can target cancer cells, release viral particles, cytokines, and their contents in the tumor cells, induce local inflammation inside the cells, and play an antitumor role. The virus uses its protein sequence to stimulate the immune system through pathogen-associated molecular patterns (Cook and Chauhan, 2020). Taking the talimogene laherparepvec system as an example, the investigators knocked out genes related to neurovirulence and antiviral evasion in VEC, a herpes simplex virus, and lead-in the single chain variable fragment of monoclonal antibody to increased talimogene laherparepvec’s target (Turkington et al., 2020). Studies have shown that the unmodified reovirus Pelareorep can also treat CRC (Goel et al., 2020). Oncolytic virotherapy can alter the tumor microenvironment and improve the efficacy of anti-programmed cell death protein 1 therapy (Ribas et al., 2017).

## 3 Metabolites, dietary, and gut microbiota in colorectal cancer

### 3.1 Metabolites

Bile acids are mainly synthesized by cholesterol in hepatocytes. Bile acids synthesized by hepatocytes are primary bile acids. Bile acids have bidirectional regulation effects on the body. At physiological concentrations, secondary bile acids have immune regulation and anti-inflammatory effects on the body, inhibiting intestinal inflammatory disease progression. High levels of secondary bile acids in the blood, bile, and feces increase the risk of cholesterol stones, damage the intestinal epithelium, and induce excessive proliferation of undifferentiated cells, leading to a premalignant state.

The metabolites of certain gut microorganisms contain secondary bile acids and SCFA, which play a role in cell proliferation. These microbial metabolites can promote colonic cell proliferation at low concentrations and inhibit cell proliferation at high concentrations. Bile acids, although synthesized in the liver, have direct or indirect antibacterial effects, thereby modulating the composition of the microbiota, which in turn regulates the size and composition of the bile acid pool (Liu et al., 2020). Patients with liver diseases such as fatty liver, fibrosis, cirrhosis, and hepatocellular carcinoma frequently exhibited intestinal dysbiosis characterized by a significant increase in aerobic and pro-inflammatory bacteria such as *Enterobacter*, *Enterococcus*, and *Clostridium*, which can accelerate production from secondary bile acids. Cholalic acid-fed mice, with a significant decrease in Firmicutes, the primary SCFA producer, from the gut. Chic acid-induced micro-dysbiosis impaired intestinal barrier function and induced low-grade intestinal inflammation, activating the transcription pathway’s IL-6 signal transducer and activator and promoting tumor progression (Wang et al., 2019). Increased bile acid levels induced by a high-fat diet promoted an increase in 7 α-dehydroxylated bacteria and increased secondary bile acids with tumor-promoting activity in the colon, particularly deoxycholic acid (Ocvirk and O'Keefe, 2017). Studies have shown that members of the *Lachnospiraceae Family* (*Phylum Firmicutes*, *Class Clostridia*) were significantly increased in the gut microbial community of the ursodeoxycholic acid-treated group (Figure 1). Bile acids bind to the receptor to regulate organisms' physiological response (Winston et al., 2021). Ursodeoxycholic acid can activate farnesoid X-activated receptor and Takeda G-protein coupled receptor 5 receptors and regulate the host’s innate immune response (Winston et al., 2020). Obercholic acid, a novel farnesoid X-activated receptor agonist, combined with the β-catenin inhibitor nitazoxanide, can inhibit CRC progression (Yu et al., 2021).

### 3.2 Dietary

The species and abundance of gut flora varied between CRC and healthy individuals. The intestinal flora is closely linked to food intake. Young women with high consumption of sugary drinks were substantially associated with increased publication rates of colorectal adenomas (particularly rectal adenomas) (Joh et al., 2021). Mice fed a high-fructose diet exhibited increased intestinal permeability, a lower proportion of *Bacteroidetes* in the gut microbiota, and a significantly increased proportion of *Proteobacteria* susceptible to colorectal tumors; diet feeding with fructose removal and supplementation could reverse tumor progression (Do et al., 2018; Nishiguchi et al., 2021). Starch-rich diet can increase the sulfur mucin content in the intestine and have a protective effect on the intestinal mucosa (Gabrielli and Tomassoni, 2018). The gut microbiota can ferment dietary fiber to produce SCFA, such as acetate, propionate, and butyrate, essential for maintaining intestinal homeostasis and intestinal epithelial cell health (Wu et al., 2018).

Regarding protein, the intake of red meat and industrially processed meat raises the risk of CRC in people (Farvid et al., 2021). In a pooled analysis of data from extensively prospective studies of Japanese men and women, meat subtype and sex were associated with CRC risk, and increased beef consumption was associated with an increased risk of CRC (women) and distal CRC (men) (Islam et al., 2019). Heme iron is a crucial component of red meat, and dietary heme influences the microbiota composition and its diversity, thus leading to dysbiosis. A heme diet reduced the gut flora α-diversity and altered the gut microbiota composition, with reduced *Firmicutes* and increased *Proteobacteria* (Seiwert et al., 2020).

A high-fat diet will increase the dietary fat in the intestine, and the intestinal dietary fat can further regulate the intestinal flora by screening fatty acid degradation genes and replacing the compound medium with the fat medium, significantly reducing the overall abundance of *Clostridium* (Budu et al., 2022). Microdysbiosis can activate the monocyte chemoattractant protein-1/C-C motif chemokine receptor 2 axis, promote the recruitment and polarization of M2 tumor-associated macrophages, and reduce SCFA content, leading to intestinal tumor cell proliferation (Martin-Gallausiaux et al., 2021). A high-fat diet increases the bile acid levels in the body, stimulates secondary bile acids levels with tumor-promoting activity in the colon, activates the bile acids-farnesoid X-activated receptor axis, stimulates the colon, and increases Wnt family member 2 B expression in fibroblast cells, thus promoting the formation of tumor immunosuppressive microenvironment (Zeng et al., 2019).

Furthermore, trace elements in the diet, such as iron, selenium, magnesium, and calcium, can regulate intestinal microbes and affect the occurrence or progression of CRC. Iron-deficiency anemia is a common complication of CRC. Studies have shown that in patients treated with oral iron, their extratemporal microbiome is rich in *Bacteriaceae* and *Bacteroides*. In contrast, the intratumoral microbiome is rich in *Nocardiaceae*, *Intrasporangiaceae*, and *Brevibacteriaceae families* and *Prevotella 9*, *Nocardioides*, *Kocuria*, *Brevibacterium*, *Veillonella*, and *Catenibacterium genera* (Phipps et al., 2021). Populations with a high *in vivo* selenium status have a lower risk of CRC. Selenoproteins can affect various signaling pathways related to the pathogenesis of CRC, and play antitumor effects (Peters et al., 2018). Studies demonstrated that selenium can modulate the intestinal flora and that the intestinal flora can also influence the absorption of selenium. Intestinal flora can produce selenomethionine from metabolizing biological selenium compounds (Peters and Takata, 2008; Takahashi et al., 2017). Magnesium intake was associated with the risk of colorectal adenoma and CRC. Studies showed that high magnesium intake may reduce the incidence of CRC in women (Larsson et al., 2005). During short-term magnesium deficiency, the content of *Bifidobacteria* and *Lactobacilli* in the intestine is low, leading to increased intestinal permeability and inflammation. Mice that were fed a regular magnesium-containing diet showed no signs of inflammation in the intestine (Pachikian et al., 2010). Calcium is an effective chemopreventive agent for colorectal adenoma (Huang et al., 2020; Emami et al., 2022). Calcium consumption can reduce the risk of death in CRC patients (Yang et al., 2019). Studies have shown that a low calcium diet can lead to intestinal microbiota dysbiosis, and high calcium supplement can restore the ecological imbalance of intestinal flora, increase the abundance of *L. reuteri*, *Lactobacillus plantarum*, *Firmicutes*, *Lactobacillus bulgaricus*, S*treptococcus thermophilus*, and *Lactobacillus*, and play an antitumor role (Gomes et al., 2015; Hua et al., 2019).

## 4 Conclusion

This paper summarized the complex interactions between antitumor agents and the gut microbiota in CRC (Figure 1). Clinicians and clinical pharmacists must realize that antineoplastic drugs affect the types and abundance of microorganisms in the gut microbiota; gut microbes can also change the effects of drugs, leading to impaired health outcomes. Simultaneously, the discipline of pharmaceutical microbiology is emerging, and clinical trials in this area have already underway. Understanding how the microbiota metabolizes drugs or improves the efficacy of anticancer therapy will significantly impact the clinical practice of drugs and generate novel ideas for regulating the intestinal microbiota and enhancing the efficacy of drug therapy.

## Abbreviations

ACEI, angiotensin I-converting enzyme inhibitors; ARB, angiotensin II receptor blockers; CRC, colorectal cancer; IGF1, Insulin like growth factor 1; IL, interleukin; NSAIDs, nonsteroidal anti-inflammatory drugs; SCFA, short-chain fatty acids.
